# An open-label non-inferiority randomized trail comparing the effectiveness and safety of ultrasound-guided selective cervical nerve root block and fluoroscopy-guided cervical transforaminal epidural block for cervical radiculopathy

**DOI:** 10.1080/07853890.2022.2124445

**Published:** 2022-09-26

**Authors:** Xiaohong Cui, Di Zhang, Yongming Zhao, Yongsheng Song, Liangliang He, Jian Zhang

**Affiliations:** a Department of Anesthesiology, Harbin Orthopedics Surgery Hospital, Harbin City, Heilongjiang Province, China; b Department of Anesthesiology, Nanchong Central Hospital, Nanchong City, Sichuan Province, China; c Department of Pain, Beijing Xuanwu Hospital, Capital Medical University, Beijing, China; d Department of Anesthesiology, Cancer Hospital of Harbin Medical University, Harbin City, Heilongjiang Province, China

**Keywords:** Selective nerve root block, transforaminal epidural steroid injection, cervical spine radiculopathy, ultrasound-guided, fluoroscopy-guided

## Abstract

**Object:**

To compare therapeutic efficacy and safety of ultrasound (US)-guided selective nerve root block (SNRB) and fluoroscopy (FL)-guided transforaminal epidural steroid injection (TFESI) for cervical spine radiculopathy (CSR).

**Method:**

156 patients with CSR randomly received US-guided SNRB verified by FL or FL-guided TFESI. We hypothesised that the accuracy rate of contrast dispersion into epidural or intervertebral foraminal space in the US group was not inferior to that in the FL group with a margin of clinical unimportance of −15%. Pain intensity assessed by Numeric Rating Scales (NRS) and functional disability estimated by neck disability index (NDI) were compared before treatment, at 1, 3 and 6 months after the intervention. Puncture time and complication frequencies were also reported.

**Results:**

88.7% and 90.3% accuracy ratings were respectively achieved in the US and FL groups with a treatment difference of −1.6% (95%CI: −9.7%, 6.6%) revealing that the lower limit was above the non-inferiority margin. Both NRS and NDI scores illustrated improvements at 1, 3 and 6 months after intervention with no statistically significant differences between the two groups (all *p* > .05). Additionally, shorter administration duration was observed in the US group (*p* < .001). No severe complications were observed in both group.

**Conclusion:**

Compared with the FL group, the US group provided a non-inferior accuracy rate of epidural/foraminal contrast pattern. For the treatment of CSR, the US technique provided similar pain relief and functional improvements while facilitating distinguishing critical vessels adjacent to the foramen and requiring a shorter procedure duration without exposure to radiation. Therefore, it was an attractive alternative to the conventional FL method.

**Key messages**
- We conducted a prospective, open-label, randomised and non-inferiority clinical trial to estimate a hypothesis that the precisely accurate delivery through ultrasound (US)-guided cervical selective nerve root block (SNRB) was non-inferior to that using FL-guided transforaminal epidural steroid injection. Additionally, US-guided SNRB was as effective as FL-guided TFESI in the treatment effect on pain relief and function improvements. Notably, the US technique might be an alternative to the conventional FL method due to the ability to prevent inadvertent vascular puncture (VP) and intravascular injection (IVI) with a shorter administration time and absence of radiation exposure.

## Introduction

Cervical spine radiculopathy (CSR) is a common disorder that has significant negative impacts on a patient’s physical functioning, mental health and social participation. The incidence ranges from 1.21 to 5.8 per 1,000 person-years from four medium to high-quality studies with prevalence values of 1.14% and 1.31% for males and females, respectively [1]. A large epidemiological study applied wide criteria encompassing neck and arm pain which was proved by corresponding MRI findings and estimated that the yearly prevalence peaked in the fourth and fifth decades [2]. Radiculopathy refers to both radicular pain and radiculopathy that can occur due to nerve root compression by disc herniation, spondylosis or a combination of both disorders [3]. C5–C7 are the most commonly affected levels usually with common sensory symptoms of unilateral dermatomal tingling or numbness and less common motor symptoms [4]. Since the risk-benefit ratio for surgery is less favourable, several systematic reviews and contemporary international treatment guidelines recommend effective conservative management strategies are preferred as first treatment option for recent onset cervical radiculopathy [5–7]. Although the evidence is limited by methodological heterogeneity and lack of comparison to a true placebo group, cervical epidural steroid injections (ESIs) are among the most common interventional pain procedures conducted for CSR. The therapeutic mechanism is to achieve a high concentration of the treating agent within the epidural space to inhibit inflammation and reduce nociceptive afferent signalling. When appropriate radiographic guidance is used, it not only provides a fairly effective short-term therapeutic effect but also offers relative safety to avoid the possibility of spinal cord infarction and spinal epidural haematoma [8]. Using a transforaminal route of injection rather than an interlaminar route is due to the rationale that injectate is delivered directly onto the site of radiculopathy and increased distribution to the ventral epidural [9]. A systematic review and meta-analysis revealed that approximately 50% of patients with cervical radicular pain due to disc herniation or degenerative spondylosis experienced ≥50% pain reduction at short- and intermediate-term follow-up after fluoroscopically guided cervical transforaminal epidural steroid injection (TFESI) [10]. However, it has been demonstrated that foraminal vertebral artery covering correlated with the severity of foraminal degenerative narrowing, oblique fluoroscopic technique suggests needle trajectory intersection with the vertebral artery in 39% of foramina. Therefore, it is usually performed without incident but potential major adverse events including spinal cord infarction due to intravascular injection (IVI) and serious neurologic injury can also occur [11,12]. Recently, cervical selective nerve root block (SNRB) under ultrasound (US) guidance has been proposed as an alternative to fluoroscopy (FL)-guided TFESI, because the proceduralist enables better visualisation of the nerve root, avoidance of abnormally situated blood vessels in real-time guidance with the absence of radiation exposure [13].

To our knowledge, far too little attention has been paid to this new technique, and there is a paucity of adequate power in estimating that US guidance is a non-inferior alternative to the FL method in the accuracy of injectate dispersion, the effectiveness of pain relief as well as physical function improvement and safety of avoiding vascular puncture (VP) or IVI. Hence, we provided detail about cervical SNRB under US guidance compared with cervical TFESI under FL guidance to report on clinical outcomes for the treatment of a large group of patients with CSP who were refractory to conservative therapy in the present open-label, non-inferiority, and randomised study.

## Methods

We conducted a randomised, open-label, non-inferiority trial with the close collaboration of the department of pain at Cancer Hospital of Harbin Medical University and Harbin Orthopaedics Surgery Hospital between January 1, 2020 and July 31, 2021. The study protocol was reviewed and approved by the Institutional Ethics Committee and review board of Cancer Hospital of Harbin Medical University and Harbin Orthopaedics Surgery Hospital and registered in the Chinese Clinical Trial Registry (ChiCTR2200055630). Written informed consent was obtained from each patient recruited in the study.

## Patients

Consecutive patients aged at least 18 years old who visited our pain clinic for neck and arm pain were screened for enrolment. Eligible patients complained of the following criteria: (1) diagnosed as cervical radiculopathy based on clinical signs and symptoms confirmed by a positive Spurling test, Arm squeeze test and Cervical distraction test; (2) clinical manifestations were identified by computerised tomography (CT) or magnetic resonance imaging (MRI); (3) no pain improvement after conservative treatments including physiotherapy, collar, traction and oral medication for at least one and half months; (4) pain intensity ≥4 on the Numeric Rating Scales (NRS); (5) invasion ≤ two nerve roots; (6) willing to undergo assignment and continue assessments. Exclusion criteria for the study were a severe cardiopulmonary failure; psychiatric disease; coagulation dysfunction; rheumatic/scoliosis/trauma injury-associated cervical radiculopathies; cervical myelopathy; known allergy to contrast media; systemic use of corticoids; injection of steroids into epidural space within 3 months; history of cervical spine surgery and pregnancy/lactation.

## Randomisation and study design

A total of 156 patients were randomly assigned in a 1:1 ratio with permuted blocks of four using randomisation list generated by Stata Version 14 (StataCorp LP, College Station, TX, USA) to receive either cervical SNRB under US guidance (US group) or cervical TFESI under C-arm guidance (FL group). The study was an open-label, pain physicians performing the procedures and all patients could not be blinded to treatment assignment because of the inherent characteristics of image guidance equipment. Two specially trained trial staff who assessed the clinical outcomes during the follow-up period and two experienced reviewers who graded the saved FL images were masked to intervention allocation. After verification of the final position of the puncture needle tip, all patients received 1 ml of diluted contrast material (Omnipaque 300, GE Healthcare, Carrigtohill, Co. Cork, Ireland) to confirm the contrast dispersion pattern, and this was followed by a slow injection of 2 ml of a mixture of 1 ml of 2% lidocaine, 0.5 ml of dexamethasone (5 mg/ml) and 0.5 ml of mecobalamin (1.5 mg/ml) for each patient. If pain reduction ＜50% in NRS score was observed after the initial injection, patients would receive a repeated injection with a 1-week interval during progression follow-up. If no pain relief or deterioration of pain was observed after two consecutive injections, patients were advised of consent withdrawal and surgical treatment.

## Procedure

### Ultrasound-guided cervical selective nerve root block verified by fluoroscopy

All cervical SNRBs were performed as outpatient procedures with US guidance by four senior pain surgeons with at least 5 years’ experience performing minimally invasive interventions for cervical radiculopathy. Patients were positioned in the supine position with their heads rotated slightly away from the ipsilateral side. After sterilisation, a short axial transverse image of the US was obtained by the application of a high-resolution (2–12Hz) linear probe (Wisonic Labat, Shenzhen Huasheng Medical Technology Co., Ltd., China) on the lateral aspect of the neck. The designated cervical level was marked through the unique landmark structure of the transverse process. The C7 transverse process was identified by only a prominent posterior tubercle as the reference point. The C4–6 transverse processes were confirmed by visualisation of the characteristical hyper-echoic two-humped camel sign by subsequently moving the probe upward. The optimal view of the corresponding nerve root was recognised as the hypo-echoic round-to-oval structure before the prominent posterior tubercle at the 7^th^ cervical spine or between the anterior and posterior tubercles at the 4–6^th^ cervical spine. Avoidance of vertebral artery and the vessels abnormally situating around the targeted cervical nerve root was achieved by probe manipulation in the transverse US scan with the colour Doppler model. Then a 22-gauge US needle (Nerve Block Needle, Itype 22 G, Shenzhen Huasheng Medical Technology Co., Ltd., China) was subsequently advanced towards the targeted nerve root from posterior to anterior direction using an in-plane technique under the real-time US guidance (Figure 2(a)). After negative aspiration of blood or cerebrospinal fluid (CSF), 1 ml of the contrast medium was slowly injected. In order to confirm the spread pattern of the injected contrast medium, the anteroposterior (AP) and lateral images were initiated with C-arm fluoroscopy. After verifying the absence of intravascular injection, 0.2 ml of 1% lidocaine was injected and monitored for 1–2 min for the presence of acute toxicity reaction including clinical manifestations of dizziness, weakness, headaches, tachycardia, auditory changes, hypersensitivity reactions and peripheral neuropraxia. The procedure was completed after the injection of the therapeutic drug mixture.

### C-arm fluroscopic-guided transforaminal epidural steroid injection

All cervical TFEIs were performed as outpatient procedures with FL guidance by the same four senior pain surgeons who performed cervical SNRBs under the guidance of the US. Patients were placed supine with their necks slightly extended to the contralateral side. The C arm was obliquely rotated 45–60° to obtain the targeted cervical neural foramen in the oblique X-ray view. The skin entry point was disinfected and anaesthetised with 1 ml of 1% lidocaine, and a 22-gauge needle (Nerve Block Needle, Itype 22 G, Shenzhen Huasheng Medical Technology Co., Ltd., China) was carefully inserted towards the outer edge of the posterior aspect of the intervertebral foramen according to an intermittent FL guidance. The optimal placement of the needle tip was finally adjusted to contact the midportion of the pedicle in AP view and the lower third of the foramen in lateral view. After negative aspiration of blood or CSF, 1 ml of contrast medium was immediately injected under a few seconds of continuous fluoroscopy (Figure 2(b)). Contrast distribution was monitored. 0.2 ml 1% lidocaine was subsequently injected to verify the absence of the above-mentioned toxicity reaction with 2 min observation. The FL-guided TFESI procedure was completed after injection of the same drug mixture as in the US method.

### Outcome measurement and data collection

The pain severity was assessed using NRS ranging from 0 (no pain) to 10 (worst possible pain). Successful pain relief was predefined as a ≥ 50% reduction in NRS score compared with the baseline value. To estimate functional disability, the neck disability index (NDI) which was considered a reliable and valid instrument for assessing cervical spine disorders was employed. The final NDI score was acquired by adding all scores from the ten questions including seven functional activity-related, two symptom-related, and one concentration-related question. According to anatomic landmarks, the intervertebral foramen area was classified into (1) extraforaminal space defined as the area outside the lateral bisector of articular pillars; (2) epidural space defined as the area within the medial bisector of articular pillars; (3) foraminal space defined as the area between the lateral and medial bisector of articular pillars (Figure 2 (b)). After the intervention, two experienced reviewers who were blinded to the treatment arm retrospectively analysed the saved FL images which were consensually classified into one of the following predefined patterns: (1) extraforaminal; (2) epidural; (3) foraminal. The optimal contrast distribution was considered as dye dispersion reaching the foraminal space or the epidural space. The rate of satisfactory contrast penetration was predefined as (number of “epidural” type + number of “epidural and foraminal” type + number of “foraminal” type)/total number. Procedure time and side effects were also recorded.

The primary outcome was the rate of satisfactory contrast distribution in the US group compared to that in the CT group after intervention. Non-inferiority was considered as the lower limit of 95% confidence interval (CI) of the difference in the primary outcome between two groups exceeding the non-inferiority margin. The second outcomes were composed of NRS scores, NDI scores, puncture time and side effects.

Data collection was conducted before intervention (T0) at our clinic, 1 month (T30), 3 months (T90) and 6 months (T180) after intervention through telephone interviews by two specially trained researchers who were blinded to the treatment method. They consensually assessed patients’ outcomes according to our prescribed criteria.

### Statistical analysis

Non-inferiority in the rate of satisfactory contrast dispersion in the US group compared to that in the FL group was our initial hypothesis. 87.3% and 89.1% satisfactory rate of contrast dispersal pattern in US and FL groups were respectively obtained based on our pre-test consisting of 10 patients per group. A sample size of 70 patients per group was specified in advance to provide 80% power with a one-sided type I error of 2.5% to detect a margin of clinical unimportance of −15%. A total of 78 subjects per group were required allowing for a 10% of the loss to follow-up.

Statistical analysis was conducted using SPSS software, version 22.0 (SPSS Inc, Chicago, IL). Statistical significance was set at *p* < .05 with a two-tailed test. Normal, non-normal distributed continuous variables and nominal variables were respectively presented as mean ± SD (standard deviation), median ± IQR (interquartile range) and proportion. The two-tailed unpaired Mann-Whitney *U* test and Chi-Squared were respectively used to compare non-normal distributed continuous data and nominal data. After the Mann-Whitney *U* test, repeated measurement data of NRS and NDI scores were further compared using two-stage step-up multiple comparisons of Benjamini, Krieger and Yekutieli with a false discovery rate (FDR) set as 1%.

## Results

### Study patients

**Figure 1.**
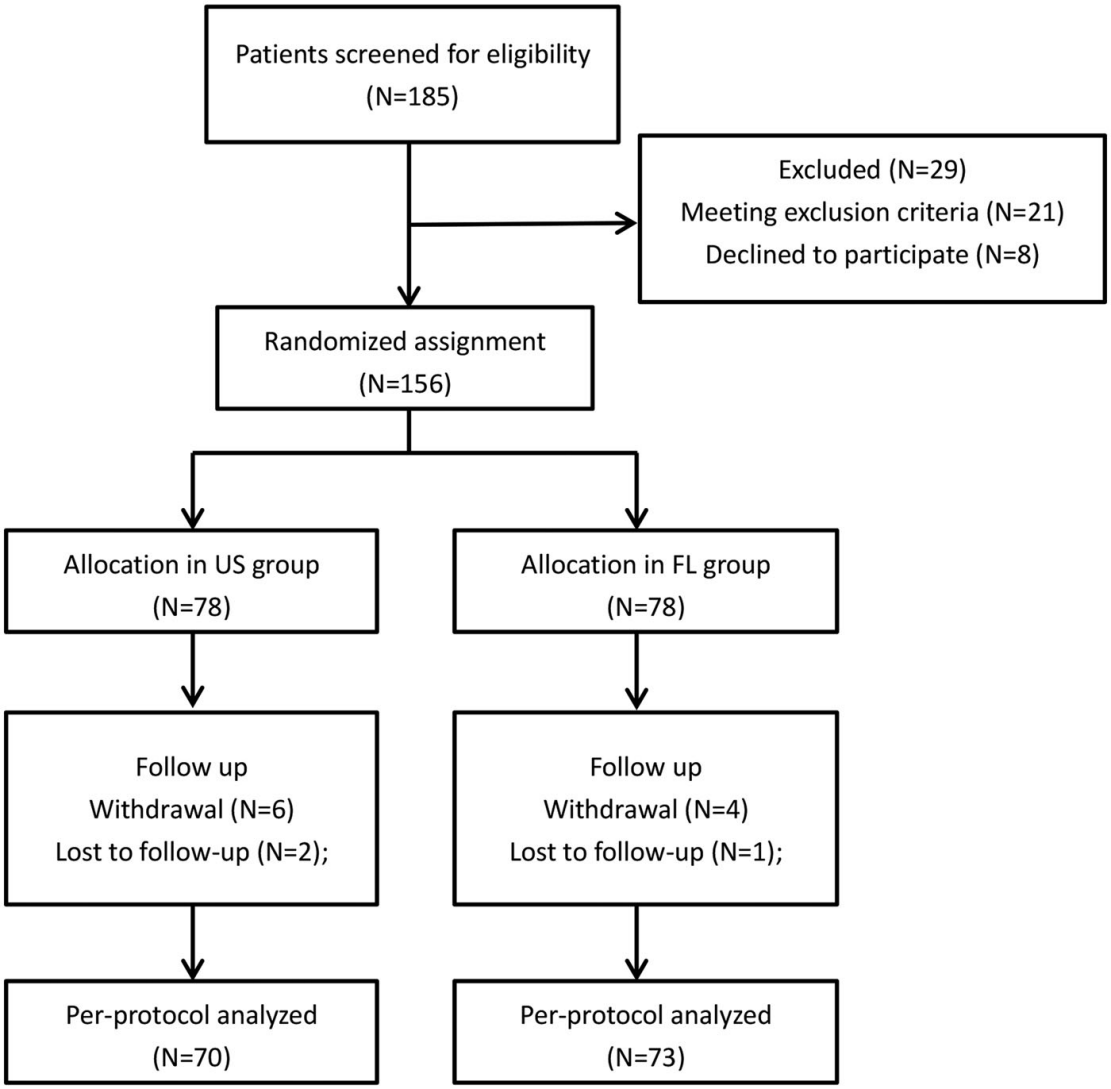
The CONSORT diagram of patient recruitment.

A total of 185 patients were enrolled in the study between January 2020 and June 2021, and 84.3% of these were randomly assigned to receive the intervention. Reasons for exclusion from randomisation and per-protocol (PP) analysis were illustrated in Figure 1 (CONSORT flow diagram). Baseline demographic data and clinical characteristic of patients were summarised in Table 1. And statistically, significant differences were not observed in the general characteristics between the two groups.

**Figure 2.**
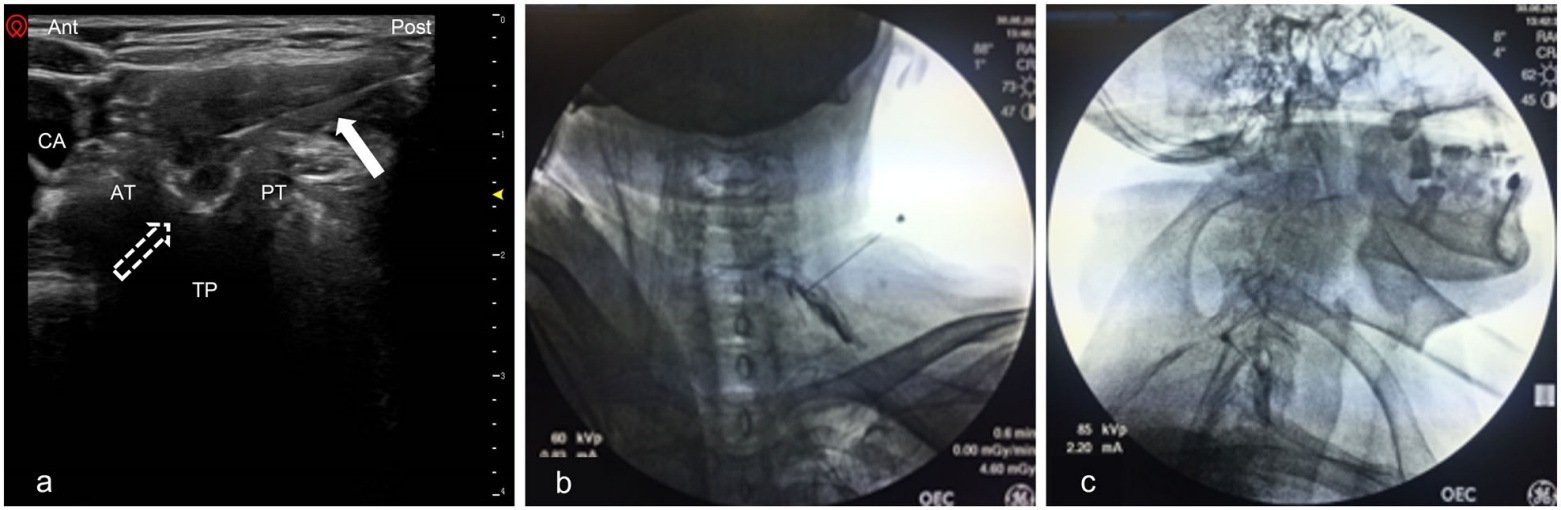
(a) The axial transverse ultrasound image of the US-guided selective nerve root block (dotted arrow) and the needle (white arrow) was advanced to target the C5 nerve toot which was between the anterior and posterior tubercle using the in-plane technique under the real-time US guidance. (b) The A-P view of the contrast media spreading into the intrafamilial space after C5 transforaminal epidural injection under FL guidance. (c) Lateral view of the contrast media which spread into the epidural space after the same C5 transforaminal epidural injection under FL guidance. US: ultrasound; FL: fluoroscopy; A-P: anterior and posterior; AT: anterior tubercle; PT: posterior tubercle; TP: transverse process; CA: carotid artery.

**Table 1.** General characteristics of patients (MEAN ± SD).

	US group (*N* = 78)	FL group (*N* = 78)	*p*
Age (years)	63.95 ± 11.67	66.10 ± 13.46	.320
Female sex, *n* (%)	40 (51.3%)	36 (46.2%)	.631
Affected side, *n* (%)			.749
Left	36 (46.2%)	39 (50.0%)	
Right	42 (53.8%)	39 (50.0%)	
VAS score at baseline	6.73 ± 1.37	6.59 ± 1.48	.555
Pain duration (months)	11.72 ± 9.95	9.89 ± 6.11	.255
Target nerve root, *n* (%)	81	86	.508
*C5*	20 (24.7%)	26 (30.2%)	
*C6*	33 (40.7%)	37 (43.0%)	
*C7*	28 (34.6%)	23 (26.7%)	
Number of injections			.380
*One injection*	58 (74.4%)	52 (66.7%)	
*Two injections*	20 (25.6%)	26 (33.3%)	
Analgesic use, *n* (%)			.403
None	23 (29.5%)	16 (20.5%)	
*NASID usage*	45 (57.7%)	49 (62.8%)	
*Weak-opioid usage*	10 (12.8%)	13 (16.7%)	

SD: standard deviation; VAS: visual analogue scale; NASID: non-steroidal anti-inflammatory drug.

### Primary endpoint

Both treatment regimens resulted in a high rate of satisfactory contrast dispersion pattern, which was respectively reported in 88.7% of cases in the US group and in 90.3% of cases in the FL group with RR = 0.845 (95%CI: 0.356, 2.006). The mean difference between both treatment arms was −1.6% (95%CI: −9.7%, 6.6%) with a non-inferiority p value equalling 0.826 (Table 2). Accordingly, the lower limit of the 95%CI of the treatment difference exceeded the non-inferiority margin of −15%, which demonstrated that the criteria for non-inferiority were not established. Therefore, US-guided cervical SNRB showed non-inferiority in the rate of satisfactory contrast dispersion into foraminal and/or epidural space, in comparison with FL-guided cervical TFESI for the treatment of CSR.

**Table 2.** Contrast dispersion patterns for US group versus FL group.

Outcome	US group (*N* = 106)	FL group (*N* = 113)	Difference in rate (95%CI)	Rate ratio (95%CI)	*χ*^2^ value	*P*
**Contrast dispersion pattern**					3.932	.140
* Epidural or/and foraminal*	46 (43.4%)	64 (56.6%)				
* foraminal*	48 (45.3%)	38 (33.6%)				
* extraforaminal*	12 (11.3%)	11 (9.7%)				
**Rate of satisfactory contrast dispersion**	94 (88.7%)	102 (90.3%)	−1.6% (−9.7%, 6.6%)	0.845 (0.356, 2.006)	0.146	.826

US: ultrasound; FL: fluoroscopy; N: number of injections.

### Secondary endpoints

**Figure 3.**
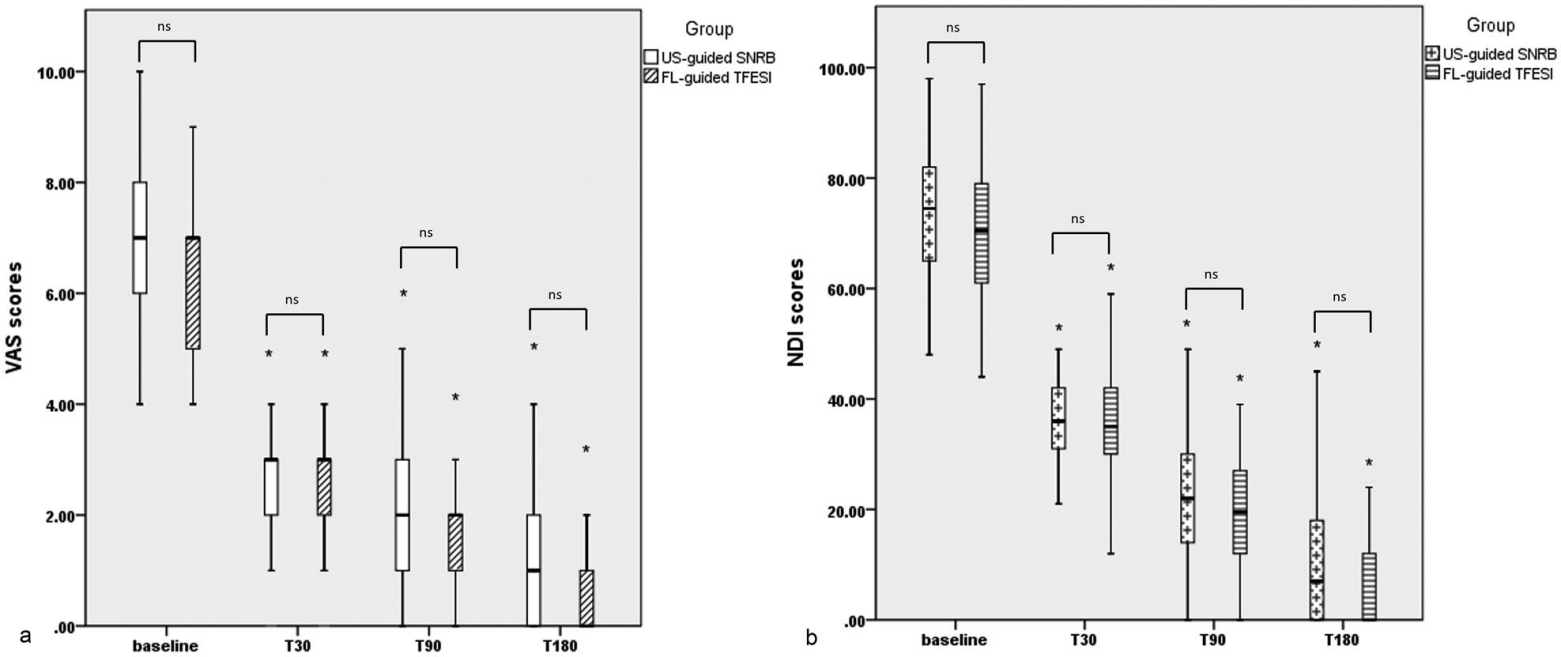
Box plots showing changes in (a) NRS scores and (b) NDI scores in two groups before and after interventions for cervical radiculopathy. ns: not significant; *multiple comparison showing *p* < .001. NRS: Numeric Rating Scales; NDI: neck disability index; US: ultrasound; FL: fluoroscopy; T30: 1 month; T90: 3 months; T180: 6 months after the intervention.

According to multiple comparisons, the median NRS scores in both two groups were significantly lower than their baseline value (all *p* < .001). However, the median in NRS scores changed from baseline to all time points during follow-up assessment did not differ statistically significantly for all patients who reported outcomes after intervention between the US group and FL group (*p* = .260 at 1 month, *p* = .108 at 3 months and *p* = .127 at 6 months post-intervention) (Figure 3). We found that at least 50% improvements in pain were reported in 82.1% and 85.9% of patients in the US group and FL group at 1 month (*p* = .663), 77.8% and 81.3% at 3 months (*p* = .684), as well as 74.3% and 76.7% at 6 months after the procedure (*p* = .864), respectively.

Both treatments improved NDI scores similarly after the intervention. The median NDI scores significantly decreased from 74.5 at baseline to 36.0 at 1 month, 22.0 at 3 months and 7.0 at 6 months during follow-up in the US group (*p* < .001). In the FL group, the median NDI score was significantly improved to 35.0 at 1 month, 19.5 at 3 months and 0.0 at 6 months post-intervention in comparison to the baseline value equalling 70.5 (*p* < .001). But there was no statistically significant shrinkage in NDI scores at all time points during the follow-up period between the two groups as shown in Figure 3.

Our result also showed that significant advantage for the US group compared with FL guidance for the reduction of procedural time, indicated by an average puncture time of 359.58 ± 124.7s in the US group versus 223.6 ± 81.9s in the FL group (*p* < .001).

### Safety

The table showed the frequency of complications after the intervention. Inadvertent avascular puncture with the manifestation of blood aspiration during puncture was nearly half as frequent in 3.8% of injections in the US group (13.8% in the FL group, *p* = .010). The risk of adverse events related to transforaminal steroids and local anaesthetics such as dizziness, nausea, vomiting and facial flushing was lower in the US group in comparison to that in the FL group, however, no statistical difference was observed between the two groups (9.4% vs. 15.9%, *p* = .062). But all these adverse reactions were resolved within 30 min after oxygen treatment in patients. No distribution of serious side effects including spinal infarction, visible haematoma and motor deficit was observed in both groups.

## Discussion

This randomised trial provided reliable data that enhanced the low-quality evidence available for a simple technique of US-guided cervical SNRB and outlined its non-inferiority in the accuracy of injectate dispersion into the foramen and epidural space compared with conservative FL-guided cervical TFESI. Our results indicated that similar improvement in pain relief and functional disability was achieved with both techniques. However, clear advantages of the US method were found regarding less risk of intravascular injection, shorter puncture time and no exposure to radiation.

Cervical radiculopathy is usually caused by cervical disc herniation, spondylosis or a combination of herniation and spondylosis which may trigger local ischaemia and inflammation [14]. Recent studies have demonstrated a favourable natural course of CSR within the first 4–6 months with complete recovery ranging from 24 to 36 months in approximately 83% of patients [15]. Except for waiting for remission by natural history, the use of conservative therapies which consists of physiotherapy, collar, traction and oral medication shows promising results at short-term follow-up [5]. For patients whose neck and radicular pain are failure to 4–6 weeks of the above-mentioned options, intensive conservative treatment should be provided in the absence of a clear indication for surgery. As the most non-surgical intervention described in previous literature, an epidural steroid injection is used frequently worldwide with many reviews reporting the success of this method for pain relief from radicular symptoms [16,17], on the basis of several mechanisms, for example, attenuating the production of prostaglandins and phospholipase A2, inhibiting their inflammatory effect and ability to ectopic discharge from unmyelinated C fibre and injured nociceptive fibre, increasing the blood flow to ischaemic nerve roots and diminishing central sensitisation [18]. In addition, mecobalamin in combination with steroids and LA may improve nerve conduction to enhance clinical therapeutic for peripheral neuropathy [19]. The transforaminal epidural injection has been postulated to be more effective than interlaminar epidural injection based on the advantage of accurate delivery to the site of pathology including the anterior ventral epidural space, dorsal root ganglion and nerve root [20]. Christina L et al. conducted a prospective clinical study to evaluate the dispersal pattern of injectate after a gadolinium-enhanced cervical epidural steroid injection *via* an interlaminar approach under fluoroscopic guidance for the treatment of CSR. All study participants underwent a cervical spine MRI within 15 min after injection. The injectate was reported to respectively disperse a mean of 8.11 and 6.63 cm in the cranial and caudal direction with 360°Circumferentially [20]. Another research reported that unilateral and ventral flow occurred in only 51% and 28% with the interlaminar approach, and the occurrence of ventral flow enhanced to 44.6% with injectate volumes ranging from 2 to 4 ml [21]. To our knowledge, the flow of injectate can be potentially blocked to the corresponding sites due to disc herniation, foraminal stenosis and epidural fibrosis. On the contrary, the transforaminal approach can better target the site of pathology in the spine, and therefore it can be advocated to replace an interlaminar route as the initial procedure in the pursuit of more accurate delivery as well as improved efficacy for unilateral CSR. As expected, according to our results, contrast dispersion was largely epidural or/and foraminal (56.6%), foraminal (33.6%) as opposed to extraforaminal (9.7%) after FL-guided TFESI in 78 patients with 113 blocks using the anterolateral approach. Moreover, 63.7% of the injections led to a reduction in the pain rating of >50% at 1-week assessment after the intervention. Image guidance usually plays an important role in the administration of cervical TFESI in order to obtain an effective injection without complications. Currently, conventional fluoroscopy-guided cervical TFESI is the most commonly available and established method. Recently, F.W. Ott et al. retrospectively identified 254 conventional FL-guided cervical TFESIs which were performed by an experienced neuro-radiologist *via* an anterolateral approach from 2011 to 2018. 75.2% of patients reported pain improvement of >50% from baseline at 15 min post-injection, and 67.7% of patients reported >50% pain scale reduction or alleviation from paresthesia at least 2 weeks after the procedure [22]. Although successful criterion varies from study to study, recent literature revealed that approximately 50% of patients experience ≥50% pain reduction at short- and intermediate-term follow-up after cervical TFESI [10]. FL-guided efficacy rates ranged from 47% to 63% [23]. Moreover, a meta-analysis suggested that A retrospective study was conducted on 28 consecutive patients diagnosed with radicular pain caused by cervical dis disease or spondylosis to evaluate repeated cervical TFESI. The average NRS score pre-intervention was 7.8 (range: 5–10) which changed to 2.9 (range: 1–7) at 3 months and 4.6 (range: 2–7) at 12 months. Patient satisfaction was 71% at 3 months and 50% at 12 months. The average system-free duration after the intervention was 7.8 months (range: 1–12) [24]. A recent observational study prospectively gathered data from 309 patients who reported greater than 12 weeks of pain with moderate to severe disability, despite conservative treatment, due to 1 or 2 level cervical degenerative herniated disc and foraminal stenosis. 72% of these patients did not receive surgical treatment and underwent cervical TFESI with the assistance of fluoroscopy, and they showed a significant decrease in pain and functional disability during the 1-year follow-up period (*p* < .05) [25]. Eric L Lin et al. reported that 70 patients who were suffering from radicular pain from a herniated cervical disc were offered surgical treatment but given the option of a cervical TFESI. It appeared that a large percentage of patients (63.5%) obtained relief of their symptoms with a good/excellent result per Odom criteria and avoided surgery for the follow-up period up to 1 year [26]. Although there has been sparse literature in reference to cervical TFESI, it suggests benefits for neck pain or/and radicular pain caused by a cervical herniated disc and non-traumatic spondylosis. Consistent with the above studies, in the present study, the median of NRS scores in the FL group was significantly lower than the baseline value [7] at 1 month [3], 3 months [2], and 6 months (0) (all *p* < .001). The median of NDI scores decreased significantly from 70.5 at baseline to 35.0 at 1 month, 19.5 at 3 months and 0.0 at 6 months after FL-guided TFESI *via* an anterolateral approach (all *p* < .001).

On the other hand, the decision to proceed with cervical TFESI not only requires comprehending of the benefits but also the risks of catastrophic or permanent adverse events. A multicenter study illustrated that 1340 anonymous surveys were sent to all U.S. physician members of the American Pain Society, and the overall response rate was 21.4%. In all, 78 complications were reported including 16 vertebrobasilar brain infarctions, 12 cervical spinal cord infarctions, and 2 combined brain/spinal cord infarctions [27]. Additionally, an analysis of 1036 fluoroscopically guided cervical TFESIs in 844 consecutive subjects reported that minor complications occurred in only 1.66% of patients which included headache/dizziness (0.59%), transient pain or weakness (0.71%), hypersensitivity reaction (0.12%), transient global amnesia (0.12%), vasovagal reaction (0.12%), and wrong site injection (0.36%) [28]. To avoid the complications which is usually due to inadvertent intravascular injection, modified recommendations are provided as the following when performing a cervical TFESI: (1) injection of contrast medium under real-time fluoroscopy (RTF) or digital subtraction angiography (DSA) [29]; (2) using 0.2 ml of 1% or 2% lidocaine for test dose to confirm the absence of local anaesthetic toxicity reaction including confusion, auditory changes, peri-oral numbness and metallic taste. In the present study, inadvertent intravascular puncture was observed in 13.8% of injections in the FL group. Transient adverse events which resolved within 30 min after an injection such as dizziness, nausea, vomiting and facial flushing occurred in 15.9% of patients in FL group. No serious and permanent side effects were observed.

Although cervical TFESI are standardly aided by FL guidance, this technique is time-consuming with exposure to radiation. On the contrary, the ability of the US is radiation-free for both patients and personnel who perform the procedure while providing detailed anatomic visualisation of soft tissues, nerves and vascular structures to potentially improve puncture precision and safety with high-resolution images in real-time [30]. Therefore, US is particularly beneficial for the management of pain in the cervical spine where a multitude of vulnerable vessels, peripheral nerves and vital soft tissues are confined to a small area [31]. Samer N Narouze et al. reported a simple technique that the feasibility of US in cervical nerve root injections with fluoroscopic confirmation. They were able to locate the needle within no more than 5 mm from the target point. And critical vessels adjacent to the foramen in the pathway of a needle trajectory could be identified using the real-time ultrasound image to avoid injury to such vessels or inadvertent intravascular injection which was the leading cause of the reported complications [32]. US-guided cervical SNRB for the treatment of CSR has been investigated by many researchers. Yongbum Park et al. conducted a retrospective analysis on 162 patients who respectively received SNRBs under the guidance of US and FL-guided TFESIs for lower cervical radicular pain. The verbal numerical scale (VNS) and DNI improved 3 months after the intervention and continued to improve until 12 months for both groups. The proportion of patients with successful treatment was illustrated as 62.5% in the US group and 58% in the FL group at 12 months. In addition, statistical differences were not observed in changes in VNS, NDI and effectiveness between the two groups [33]. Haemi Jee et al. compared the short-term effects and advantages of US-guided SNRB with FL-guided TFESI for radicular pain in the lower cervical spine through a randomised blinded controlled study. They found VNS improved 2 weeks and 12 weeks after the intervention in both groups. And there were no statistical differences in VNS, NDI and effectiveness between groups. In 35% of patients at the US, vessels were identified at the anterior aspect of the foramen, 18.3% of patients had a critical vessel at the posterior aspect of the foramen and 8.3% of patients had an artery continue medially into the foramen. In all cases, these vessels might well have been in the pathway of the puncture if positioned under FL guidance. 5 cases of intravascular injections were observed only in the FL group without significant difference between the groups [34]. In a recent comparative study, patients with radicular pain in the lower cervical spine were respectively submitted to US-guided SNRB (*n* = 44) or FL-guided cervical interlaminar epidural steroid injection (ILESI) (*n* = 41) or FL-guided cervical TFESI (*n* = 37). Both the NDI and VNS scores showed improvements at 1, 3 and 6 months after the last injection in all groups, with no significant differences among groups (*p* < .05). Blood was aspirated before injection in 7%, 14% and 0% of patients in the FL-guided ILESI, TFESI and US-guided SNRB groups, respectively. Intravascular contrast spread was respectively noted in 4.9% and 18.9% of cases in the FL-guided ILESI and TFESI groups. Therefore, compared with the other two techniques, US-guided SNRB had a low intravascular injection rate and required a shorter administration duration while providing similar pain relief and functional improvement [34]. Although in this technique, the needle tip only reaches the nerve root, the contrast medium did not mainly spread to the extraforaminal space. According to our results, we performed a total of 106 US-guided SNRB with fluoroscopic confirmation. The contrast medium mainly spread into intervertebral foraminal space in 45.3% of patients. There was no statistically significant difference in contrast dispersion pattern between the two groups after injection of 1 ml contrast medium. The rate of satisfactory contrast distribution which contrast dispersed into epidural space, foraminal space or a combination of both spaces was respectively reported as 88.7% in the US group and 90.3% in the FL group with RR = 0.845 (95%CI: 0.356, 2.006) as well as mean of treatment difference of −1.6% (95%CI: −9.7%, 6.6%) revealing that the lower limit was above the non-inferiority margin. Hence, it was in favour of our hypothesis that the rate of precisely accurate delivery was not inferior to that in the FL group. Our results also showed that no significant difference in the percentage of patients who reported ≥50% pain relief was found for US and FL groups at all time points during the follow-up period. And there was no statistically significant shrinkage in NRS and NDI scores at all time points during the follow-up period between the two groups (Figure 3), which demonstrated that US-guided SNRB was as effective as FL-guided TFESI in the treatment effect on pain relief and function improvements.

Consistent with the previous study, identification of critical vessels surrounding the intervertebral foramen was allowed using colour Doppler model with in-plane technique to reduce the risk of advertent VP and IVI [35]. Our results showed that under the real-time US guidance unintended VP was nearly half as frequent in 3.8% of injections in the US group (13.8% in the FL group, *p* = .010), which illustrated that the US is not 100% accurate in preventing intravascular puncture or injection. This may be related to the limitations in visualisation of bone structure and small vessels adjacent to intervertebral foramen by the US-guided method. Therefore, when choosing this technique, the proceduralist should familiarise themself with the anatomic structures in the cervical spine and be precisely experienced with the scanning and injection technique of intervention in the cervical region. However, there was no sign of major complications. Besides, the mean puncture time in the US group was significantly shorter than that in the FL group.

Several limitations were presented in the study. Firstly, it was impossible to keep patients and surgeons blinded due to the nature of the intervention. Secondly, patients could not be entirely prevented from using oral medication during the follow-up period. Thirdly, the long-term efficacy and safety should be evaluated based on a well-designed randomised controlled study in the future.

In conclusion, in the present study, US-guided SRNB was proved as a non-inferior alternative to FL-guided TFESI in the supply of precise accuracy delivery to the site of pathology to achieve similar pain relief and functional improvements. In addition, there were several advantages including the prevention of VP and/or IVI, a shorter administration time and no exposure to radiation.

## Data Availability

The data that support the findings of this study are available from the corresponding author, Professor Jian Zhang, upon reasonable request.

## Abbreviations

CSR: cervical spine radiculopathy
SNRB: selective nerve root block
TFESI: transforaminal epidural steroid injection
US: ultrasound
FL: fluoroscopy
CT: computed tomography
MRI: magnetic resonance imaging
NRS: Numeric Rating Scales
NDI: neck disability index
VNS: verbal numeric pain scale
AP: anteroposterior
RTF: real-time fluoroscopy
DSA: digital subtraction angiography
VP: vascular puncture
IVI: intravascular injection
ESI: epidural steroid injection
ILESI: interlaminar epidural steroid injection
IQR: interquartile range
CI: confidence interval
CSF: cerebral spinal fluid
